# IGAR: Indonesian government applications review for sentiment analysis dataset

**DOI:** 10.1016/j.dib.2026.112708

**Published:** 2026-03-20

**Authors:** Mahmud Isnan, Bens Pardamean

**Affiliations:** a Computer Science Department, School of Computer Science, Bina Nusantara University, Jakarta 11480, Indonesia; b Bioinformatics and Data Science Research Center, Bina Nusantara University, Jakarta 11480, Indonesia; c Computer Science Department, BINUS Graduate Program - Master of Computer Science Bina Nusantara University, Jakarta 11480, Indonesia

**Keywords:** Sentiment analysis, Government apps, Natural language processing, User review, Google Play Stores

## Abstract

The government has recently adopted mobile applications to enhance service delivery for citizens. However, these applications often generate mixed reactions among users. Many citizens express their opinions through reviews and ratings on the Google Play Store, providing valuable information for sentiment analysis. Leveraging this, the present paper introduces the Indonesian Government Application Review (IGAR) dataset, a collection of 617,722 user reviews from six popular government-related applications in Indonesia: Mobile JKN, MyPertamina, KAI, JMO, Satusehat, and BMKG. The reviews, originally written in Indonesian, were manually annotated as positive, neutral, or negative based on rating scores. Among the dataset, positive sentiment accounts for 336,449 reviews, negative sentiment with 246,898 reviews, while 34,375 reviews are categorized as neutral. To extend the usability of the dataset for broader research contexts, all reviews were translated into English and further processed using the Valence Aware Dictionary and sEntiment Reasoner (VADER) for automated sentiment labeling. Through VADER classification, 324,660 reviews were identified as positive, 173,329 as neutral, and 119,733 as negative. This dataset thus provides a valuable resource for advancing sentiment classification research using machine learning and deep learning model on government-related applications in Indonesia.

Specifications Table

**Table:** 

Subject	Computer Sciences
Specific subject area	Natural Language Processing, Sentiment Analysis, Indonesian Language, Text Classification
Type of data	Table, text.
Data collection	The data were collected from Google Play Store application page using *google-play-scraper* library based on the Python programming language. User reviews of Six Indonesian government apps were extracted such as Mobile JKN, MyPertamina, KAI, JMO, Satusehat and BMKG. Dataset labelling has been done using two different approaches: manual labelling based on user ratings and automatic labelling using VADER Sentiment.
Data source location	Country: Indonesia and dataset were collected at Bina Nusantara University.
Data accessibility	Repository name: Mendeley Data Data identification number: 10.17632/7zryc6k76z.3 [1] Direct URL to data: https://data.mendeley.com/datasets/7zryc6k76z/3
Related research article	None

## 1 Value of the Data

• The IGAR dataset provides the first-of-its-kind resource for researchers working on user sentiment analysis of government applications in Indonesia. It contains 617,722 user reviews collected from six well-known government apps: Mobile JKN, MyPertamina, KAI, JMO, Satusehat, and BMKG. This large-scale dataset offers a comprehensive view of user sentiment in real-world e-government service contexts and enables researchers to analyze sentiment across different sectors and compare patterns between them.
• The dataset is available in multiple languages. All reviews were originally written in Indonesian and later translated into English, making it a valuable resource for advancing natural language processing (NLP) research in underrepresented languages. This multilingual feature is particularly useful for researchers focusing on sentiment analysis, e-government, and localized language modeling. It also facilitates cross-lingual model evaluation and comparison, supporting studies that assess how well sentiment models generalize between Indonesian and English.
• The dataset includes reviews collected over time, allowing researchers to the changes in public perception following policy updates or app feature releases. It also supports benchmarking specific NLP tasks such as apect-based sentiment analysis and temporal trend analysis of user reviews.
• Researchers can leverage this dataset to address challenges in imbalanced classification, examine the effectiveness of data augmentation techniques, and evaluate sentiment classification performance within real-world government service applications.
• This dataset offers interdisciplinary use as it can support studies in public policy evaluation based on mobile service digital performance, particularly in assessing the effectiveness, accessibility, and responsiveness of government-related digital applications. Furthermore, it can contribute to digital governance research and user-experience analytics by providing insights into how citizens interact with and perceive public digital services. In real-world policy and operational analytics, the dataset can be used by government agencies to monitor public sentiment toward digital services in near real-time, enabling early detection of service disruptions or user dissatisfaction. This, in turn, supports data-driven decision-making and helps prioritize service improvements.

## 2 Background

E-government aims to enhance public access to government services, improve the availability of government-owned information, address societal challenges, and promote equality among all citizens [2]. In Indonesia, the adoption of e-government services via mobile applications increased by 25% in the third quarter of 2020 [3]. Despite this growth, citizen responses to government-related applications remain divided: while some users appreciate the convenience and efficiency offered, others raise concerns about usability, reliability, and overall service quality. Applications such as Mobile JKN, MyPertamina, KAI, JMO, Satusehat, and BMKG experienced an increase in user reviews, offering insight into both the effectiveness of service delivery and the challenges faced by the users. These reviews can be leveraged for sentiment analysis using Natural Language Processing (NLP) to evaluate public perceptions [4] and identify issues in government digital services [5].

This dataset was compiled to systematically analyze user reviews. Since manual analysis of such large-scale text data is inefficient and prone to bias, reviews were collected through web scraping using google-play-scraper library. They were categorized using manual annotation based on review ratings and automated labelling with Valence Aware Dictionary and sEntiment Reasoner (VADER) [6]. This data article supports research project on sentiment analysis of government-related application in Indonesian.

## 3 Data Description

The dataset comprises two main files that are publicly accessible via Mendeley Data repository. In the repository, a sample python script is also included to reproduce visualization (Data_Visualization.ipynb). Table 1 provides summary table of datasets. To enhance transparency and facilitate data traceability, Table 2 presents a mapping table that links each figure and table in this paper to its corresponding dataset file and the specific variables used. The files associated with this IGAR dataset are organized as follows:

**Table 1:** Summary of datasets.

File Name	Size	Format	Number of columns	Number of records
Rating_labeled	96MB	CSV	7	617722
VADER_labeled	107MB	CSV	12	617722

**Table 2:** A mapping table that links each figure and table in this paper to its corresponding dataset file and the specific variables used.

Figure/Table	Data Source	Relevant Attribute	Description
Table 3	Rating_labeled.csv	App, content, translation, score, at, appVersion, labelScoreBase	Description of dataset in Rating_labeled.csv
Table 4	VADER_labeled.csv	app, content, score, at, appVersion, translation, vader_neg, vader_neu, vader_pos, vader_compound, vader_label, vader_confidence	Description of dataset in VADER_labeled.csv
Table 5	Rating_labeled.csv	app, labelScoreBase	Distribution of sentiment rating-based labels for each application
Table 6	Rating_labeled.csv	app, labelScoreBase, content, translation	Sample data for each sentiment class
Table 8	VADER_labeled.csv	app, vader_label	Distribution of sentiment VADER-based labels for each application
Fig. 3	Rating_labeled.csv	app	Number of reviews for each application
Fig. 4	Rating_labeled.csv	app, labelScoreBase	Sentiment distribution for government-related application
Fig. 5	Rating_labeled.csv	Content, labelScoreBase	Word Cloud group by label sentiment from all applications in Rating_labeled.csv
Fig. 6	VADER_labeled.csv	app, vader_label	Sentiment distribution using VADER for government-related applications
Fig. 7	VADER_labeled.csv	Content, vader_label	Word Cloud group by label sentiment from all applications in English translation
Fig. 8	Rating_labeled.csv VADER_labeled.csv	labelScoreBase, vader_label	Confusion matrix of agreement labels using Cohen’s kappa

### 3.1 Manual Labelling (Rating_labeled.csv)

This file consists of 617,722 user reviews for six government-related applications Mobile JKN, MyPertamina, KAI, JMO, Satusehat, and BMKG. Each row contains structured information such as the application name, the review text, the rating score, the time the review was created, the application version, and the sentiment label. These key attributes are organized into columns, which are described in Table 3, which also contains descriptions, data types and example values ​​of each attribute. Providing reviews in a structured format is essential to enable reproducibility, facilitate preprocessing, and support a wide range of natural language processing tasks, particularly sentiment analysis, as has also been emphasized in previous dataset publication [7].

**Table 3:** Description of dataset in Rating_labeled.csv.

Column Name	Description	Data Type	Value
app	The name of the application being reviewed	Object	satusehat
content	The review text or comment written by the user	Object	Jadi lebih waspada sama bahaya covid 19
translation	English translation of the original review text	Object	So more alert to the dangers of Covid 19
score	The rating given by the user (scale 1–5)	Int64	5
at	The timestamp indicating when the review was created	Object	2021–10–14 06:02:30
appVersion	The version of the application used for the review	Object	3.4.6
labelScoreBase	The sentiment label assigned to the review	Object	Positive

### 3.2 Automated labelling (VADER_labeled.csv)

**Fig. 1:**
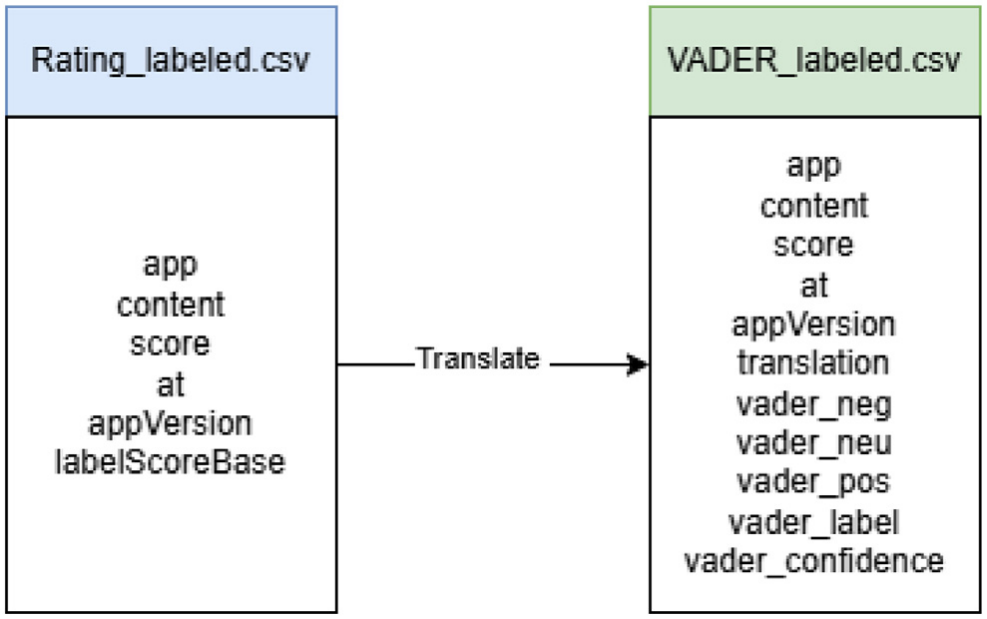
A visual schema of relationship between files (rating and VADER label).

In addition to manual labeling based on rating scores, this dataset was also enriched with an automated labeling process using the VADER Sentiment. VADER is a lexicon-based sentiment analysis method designed to detect text polarity (positive, negative, neutral) by considering emotional intensity. VADER is widely used on short texts such as app reviews [8], social media comments [9], and online news [10] because it is able to capture the nuances of everyday language, including short expressions, capital letters, excessive punctuation, and emoticons. This file extends the structure of manual labelled files by incorporating additional attributes that enhance both multilingual analysis and automated sentiment classification. Specifically, it introduces a translation column, which provides the English version of the original review text, thereby enabling cross-lingual NLP applications. Fig. 1 depicts a visual schema of relationship between files (rating and VADER labeled). Furthermore, the dataset integrates outputs from the VADER sentiment analysis model. These include probabilistic sentiment scores (*vader_neg, vader_neu, vader_pos*), the aggregated vader_compound score, the categorical sentiment classification (*vader_label*), and the associated confidence score (*vader_confidence*). The descriptions of these additional attributes and sample data are summarized in Table 4, which highlights how the extended dataset serves as both a resource for supervised learning with human-assigned labels (rating) and a benchmark for evaluating automated sentiment analysis methods.

**Table 4:** Description of dataset in Vader_labeled.csv.

Column Name	Description	Data Type	Value
app	The name of the application being reviewed	Object	satusehat
content	The review text or comment written by the user	Object	Mantap…sangat membantu
translation	English translation of the original review text	Object	Great … very helpful
score	The rating given by the user (scale 1–5)	Int64	5
at	The timestamp indicating when the review was created	Object	2021–10–14 06:01:28
appVersion	The version of the application used for the review	Object	3.4.6
vader_neg	Sentiment score for the negative polarity generated by VADER	Float64	0.000
vader_neu	Sentiment score for the neutral polarity generated by VADER	Float64	0.218
vader_pos	Sentiment score for the positive polarity generated by VADER	Float64	0.782
vader_compound	Aggregated sentiment score calculated by VADER across all polarities	Float64	0.8016
vader_label	Final sentiment class predicted by VADER (Positive, Neutral, Negative)	Object	positive
vader_confidence	Confidence score corresponding to the predicted VADER sentiment label	Float64	0.8016

## 4 Experimental Design, Materials and Methods

**Fig. 2:**
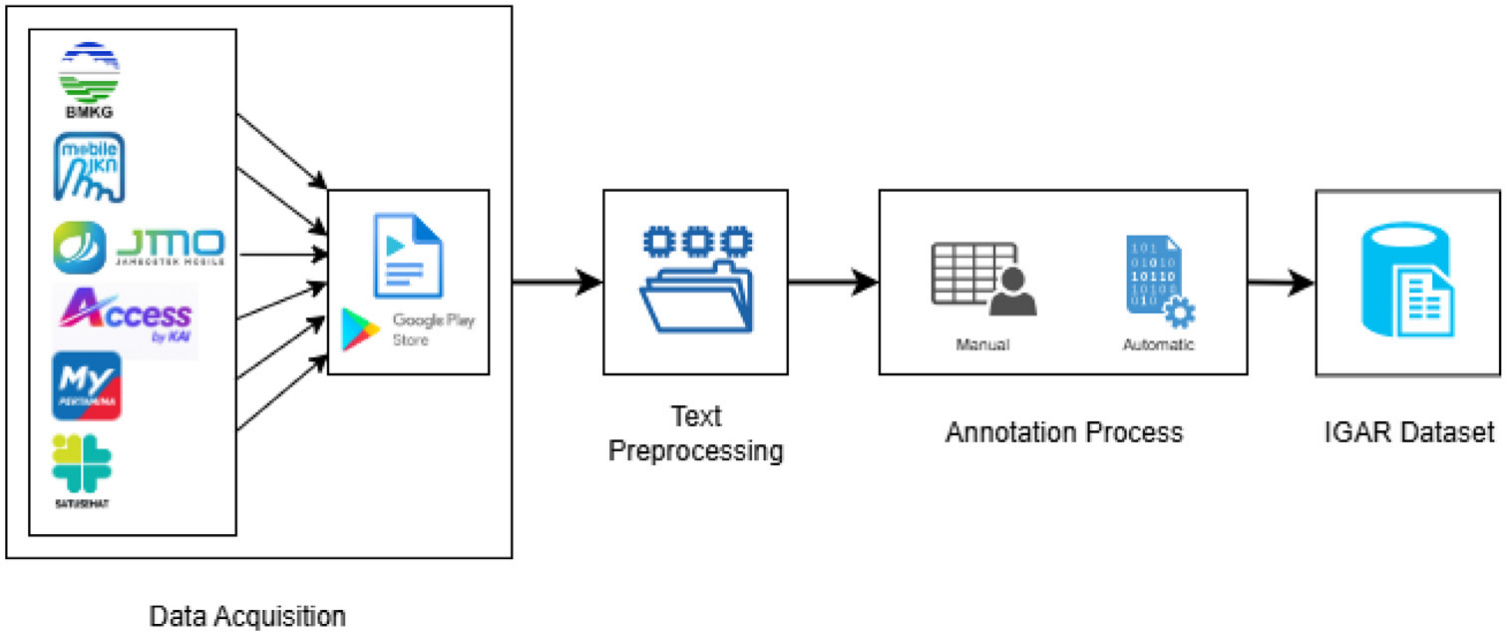
Curation process of IGAR dataset.

Fig. 2 illustrates the IGAR Dataset creation process inpired by the previous study [11], starting from the data acquisition process to producing a ready-to-use dataset. The first stage is data acquisition, where data is obtained from various public service applications available on *Google Play Store*, such as Mobile JKN, MyPertamina, KAI, JMO, Satusehat, and BMKG. This process was done on 15 September 2025. The collected data then enters the text preprocessing stage to be cleaned of unnecessary information while maintaining user privacy. At this stage, various personal elements and metadata such as *userReviewId, userName, userImage, replyContent, replyAt, thumbsUpCount*, and *reviewCreatedVersion* are removed, leaving only the relevant core review text [12].

Furthermore, the prepared data undergoes the annotation process. The annotation process is carried out in two stages: manual labeling based on user ratings, and automatic annotation using VADER (Valence Aware Dictionary and sEntiment Reasoner), a lexicon-based sentiment analysis model capable of detecting positive, negative, and neutral sentiment polarities in review text. The final result of this entire series of processes is the formation of the IGAR Dataset, a clean, well-annotated, structured dataset that is ready to be used for research and development of artificial intelligence-based systems. The collected reviews are available from 2012 to 05–11 17:18:48 to 2023–03–26 01:32:45 and all timestamps are recorded in the Asia/Jakarta time zone (GMT+7).

### 4.1 Data acquisition and preprocessing

The dataset was collected using the *google-play-scraper* library (v1.2.7) on a Windows environment running python version 3.13.5. This library enabled automated retrieval of user reviews from Android applications. This approach allowed for the systematic collection of large-scale review data, including textual content, rating scores, timestamps, and application version information. Data were retrieved from the MobileJKN ('app.bpjs.mobile'), MyPertamina ('com.dafturn.mypertamina'), KAI ('com.kai.kaiticketing'), JMO ('com.bpjstku'), Satusehat ('com.telkom.tracencare'), and BMKG ('com.Info_BMKG'). The scraping process was configured with the following parameters: lang='id' (Indonesian language), country='id' (Indonesia region), sort=Sort.NEWEST (sorted by most recent reviews), count=100,000 (maximum number of reviews retrieved), and filter_score_with=None (no filtering by rating). To ensure complete data collection, the continuation_token was used to sequentially retrieve additional batches of reviews. As illustrated in Algorithm 1, the procedure begins by specifying the application identifier, language, country, sorting order, and the maximum number of reviews to be retrieved. The function returns an initial set of reviews along with a continuation token. If the continuation token is available, it is repeatedly used to request additional reviews until no further data can be collected. However, the raw output of this process still contains personal or sensitive information such as user identifiers, usernames, and profile images. The corresponding Python implementation, demonstrating the use of key function parameters in the *google-play-scraper* library, is available in the repository under the notebook file “IGAR_Dataset.ipynb”.

**Algorithm 1:** Pseudocode of extracted user review from Google Play Store.

1: **Define** the target application based on its App ID (e.g., "com.telkom.tracencare"). 2: **Call** the review extraction function with the following parameters: - Language of reviews = Indonesian - Country = Indonesia - Sorting order = newest - Maximum number of reviews = 200,000 - Score filter = none (all ratings included) → **Output**: initial set of reviews + continuation token 3: Use the continuation token to continue retrieving subsequent reviews. → **Output**: additional set of reviews 4: **Combine** all retrieved reviews into a single dataset.

Therefore, a preprocessing step was conducted using python programming language (3.13.5) on Windows environment to remove personal users’ attributes, ensuring that the final dataset is ethically compliant and suitable for research purposes. Only relevant attributes such as the review text, rating score, timestamp, and application version were retained. This practice aligns with established ethical guidelines in data science and natural language processing, which emphasize the importance of anonymization and the removal of personally identifiable information (PII) prior to analysis [13]. Consequently, the resulting dataset is ethically compliant and research-ready, ensuring that subsequent analyses focus exclusively on content relevant to sentiment analysis without exposing sensitive user details. Missing or incomplete data were not handled or imputed during the preprocessing, and therefore some records with missing values remained in the dataset. This decision was made to preserve data integrity and allow future research to apply their own preferred missing-data handling strategies according to their research objectives.

**Fig. 3:**
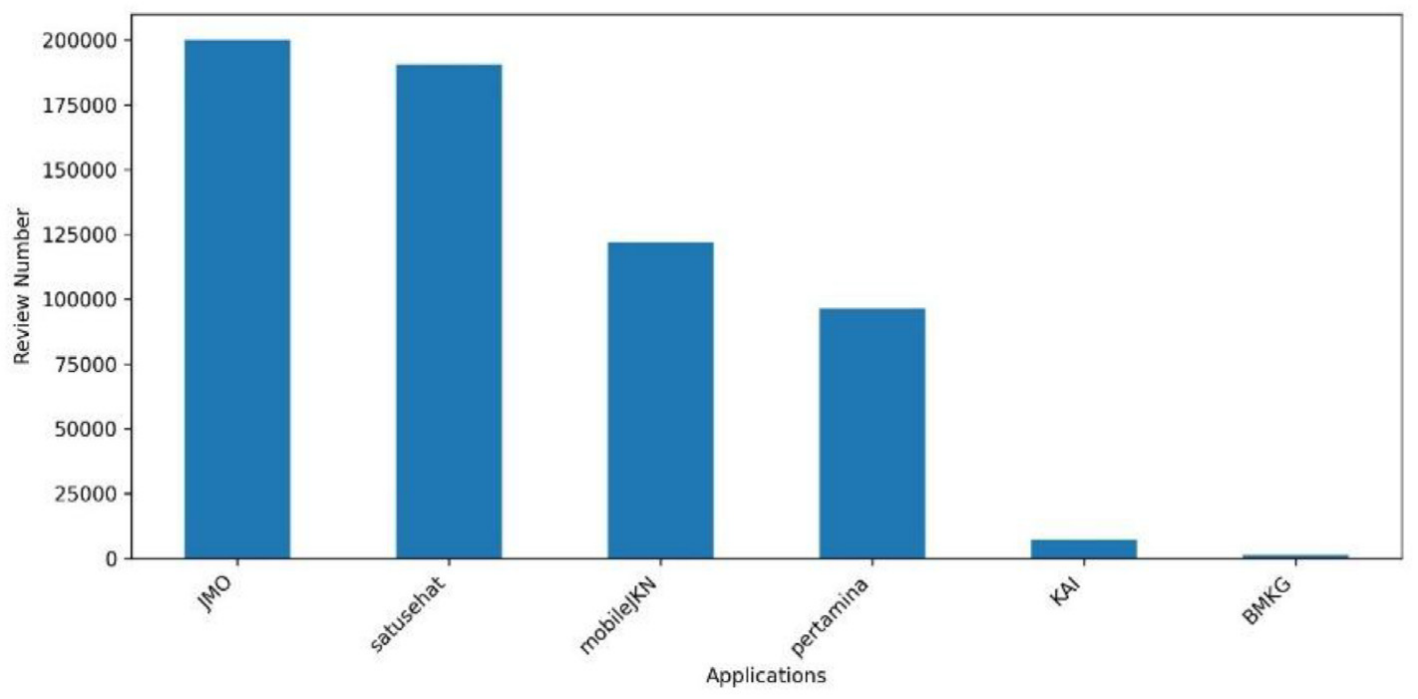
Number of reviews for each application.

Fig. 3 illustrates the distribution of extracted user reviews across different government-related applications. It can be observed that the number of reviews per application is imbalanced. This imbalance arises from the limitations of the *google-play-scraper* tool, which is unable to extract the complete set of reviews from Google Play. Consequently, the amount of data retrievable is not fixed and may vary for each application. For instance, the BMKG application actually contains >1321 reviews; however, the extraction process was interrupted at that point and could not proceed further.

### 4.2 Annotation process

First, the cleaned dataset was manually labeled based on the rating scores provided by users. In this approach, reviews with higher ratings (score 4 or 5) are generally associated with positive sentiment, while those with lower ratings (scores 1–2) are associated with negative sentiment. Ratings in the mid-range (score 3) are typically interpreted as neutral. This heuristic has been widely adopted in prior sentiment analysis studies because user ratings serve as an implicit ground truth for the polarity of textual reviews [14,15]. Compared to fully manual annotation, which is often costly and time-consuming [16], leveraging existing rating scores provides an efficient and scalable way to construct labeled datasets for supervised learning tasks. However, this method also introduces certain limitations, as the textual content of a review may not always perfectly align with the numeric score assigned by the user. Despite these challenges, rating-based labeling remains a widely accepted baseline in app review mining and sentiment classification research [17].

**Fig. 4:**
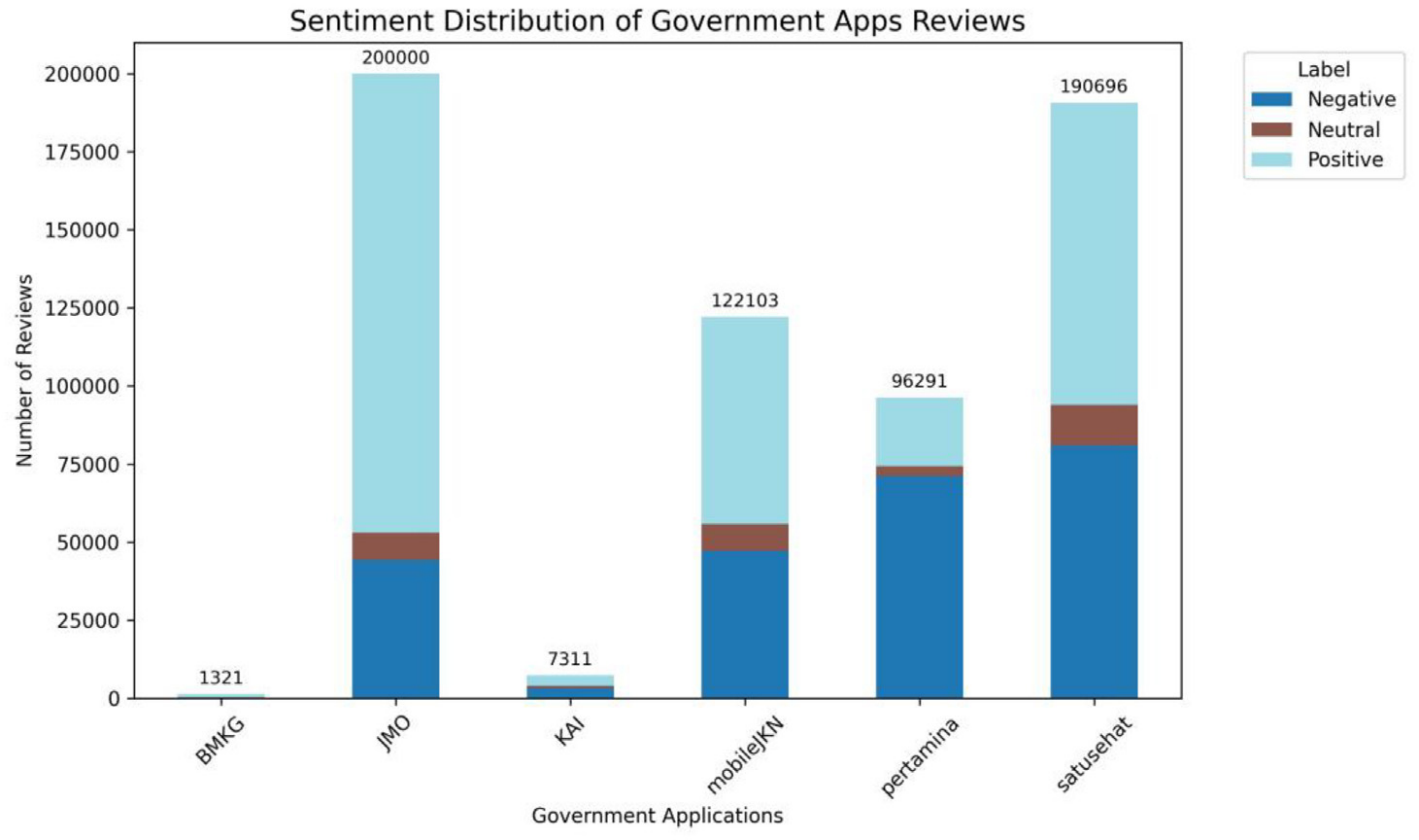
Sentiment distribution for government-related application.

Among the dataset, positive sentiment dominates with 336,449 reviews, followed by negative sentiment with 246,898 reviews, while neutral sentiment is the least represented with only 34,375 reviews. Fig. 4 illustrates the distribution of sentiment labels across applications based on user ratings (see Table 5 for details), and representative examples for each label are presented in Table 6.

**Table 5:** Distribution of sentiment rating-based labels for each application.

Government Apps	Label			Total
	Negative	Neutral	Positive	
BMKG	111	99	1111	1321
JMO	44,328	8654	147,018	200,000
KAI	3206	780	3325	7311
MobileJKN	47,182	8596	66,325	122,103
MyPertamina	71,136	3175	21,980	96,291
Satusehat	80,935	13,071	96,690	190,696
**Total**	246,898	34,375	336,449	617,722

**Table 6:** Sample data for each sentiment class.

App	Sentiment	Review in Indonesian	Translation
BMKG	Negative	Tidak bisa dibuka. Sudah ditunggu 15 menit tidak bisa diakses prakiraan cuaca nya.. Server nya belum memadai	Can't open. I've been waiting for 15 min and can't access the weather forecast. The server isn't adequate
	Neutral	Sedia payung sebelum hujan…setidaknya informasi sedikitpun kita harus dapatkan dari aplikasi ini	Prepare an umbrella before it rains…at least we should get a little information from this application
	Positive	Bermanfaat	Beneficial
JMO	Negative	Aplikasi jmo payah. Mau login aja gk bisa2 padahal sinyal kenceng. Udh versi yg terbaru tetep aja gk bisa di buka	The jmo app sucks. I can't even log in even though the signal is fast. Even the latest version still can't be opened
	Neutral	Kenapa setiap buka apk selalu permintaan update	Why every time I open the apk it always asks for an update
	Positive	Terimakasih, sangat membantu	Thank you, very helpful
MobileJKN	Negative	Tiap bulan wajib update aplikasi trs… Ribet bgt sumpah. Loading nya lambat, padahal pake simpati di kota besar. Kemahalan juga iuran perbulannya	Every month you have to update the app… It's so annoying, I swear. Loading is slow, even though I'm using Simpati in a big city. The monthly fees are also too expensive
	Neutral	Mohon di bantu dong, mau reset pasword tapi mau pake no.hp baru, soalnya yg lama udah ga aktif,. Gmana min?	Please help me, I want to reset my password but I want to use a new phone number, because my old one is no longer active. What should I do, admin?
	Positive	Mempermudah buat ganti faskes tingkat 1 terutama cocok.untuk pekerja yg sering di mutasi dalam tugas kerja	Mempermudah buat ganti faskes tingkat 1 terutama cocok.untuk pekerja yg sering di mutasi dalam tugas kerja
KAI	Negative	Aplikasi keluar sendiri	The application exits by itself
	Neutral	Pesan tiket KA jadi semakin mudah. Cuma kalau mau edit profile gimana caranya ya…????	Booking train tickets has become easier. But how do I edit my profile?
	Positive	Mempermudah mencari jadwal KAI	Make it easier to find KAI schedules

**Fig. 5:**
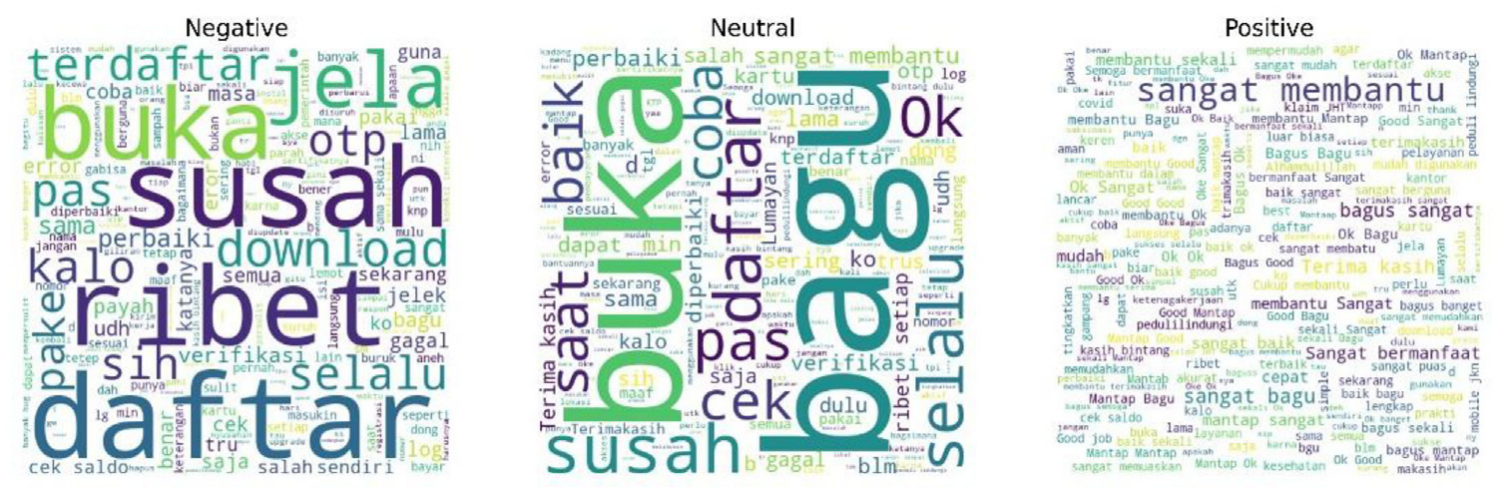
Word Cloud group by label sentiment from all applications Rating_labeled.csv.

Fig. 5 presents word cloud visualizations representing the distribution of dominant words in user reviews across three sentiment categories: negative, neutral, and positive. In the negative sentiment group, the most prominent words include susah (difficult), ribet (complicated), daftar (register), buka (open), otp, and error. These terms indicate that the majority of user complaints are related to technical issues, particularly in the registration, login, and OTP verification processes, which are the main sources of dissatisfaction.

In the neutral sentiment group, a combination of positive and negative terms appears, such as bagus (good), baik (fine), ok, susah (difficult), and cek (check). This pattern reflects the presence of mixed sentiments, in which positive and negative experiences are expressed simultaneously. Such a phenomenon supports the concept of sentiment valence, where neutral reviews often result from a cancel-out effect between positive and negative components of the user experience [18]. This category also poses challenges for sentiment analysis, especially for word-based approaches and deep learning models, as the coexistence of positive and negative words within a single review may confuse classification algorithms and reduce prediction accuracy [19]. Consequently, neutral sentiment requires more sophisticated approaches, such as aspect-based sentiment analysis or contextual representation methods.

In the positive sentiment group, dominant words include sangat membantu (very helpful), bagus (good), mantap (excellent), baik (fine), cepat (fast), and terima kasih (thank you). The prevalence of these words illustrates a high level of satisfaction, where the application is perceived as useful, easy to use, and providing a pleasant user experience.

Prior to applying VADER, all user reviews were translated into English using Google Translate through the *deep-translator* library (v1.11.4) to ensure compatibility with the VADER lexicon. Once translated, each review was processed by VADER, which generates a compound score ranging from −1 to +1. The implementation of VADER used in this study was the one included in the *NLTK package* (v3.9.1) with default configuration. This score reflects the overall polarity of the text, where values closer to –1 indicate strongly negative sentiment, and values closer to +1 indicate strongly positive sentiment, as illustrated in Table 7.

**Table 7:** The typical threshold for sentiment class.

Label	Compound score
Negative	≤−0.05
Neutral	>−0.05and<0.05
Positive	≥0.05

**Fig. 6:**
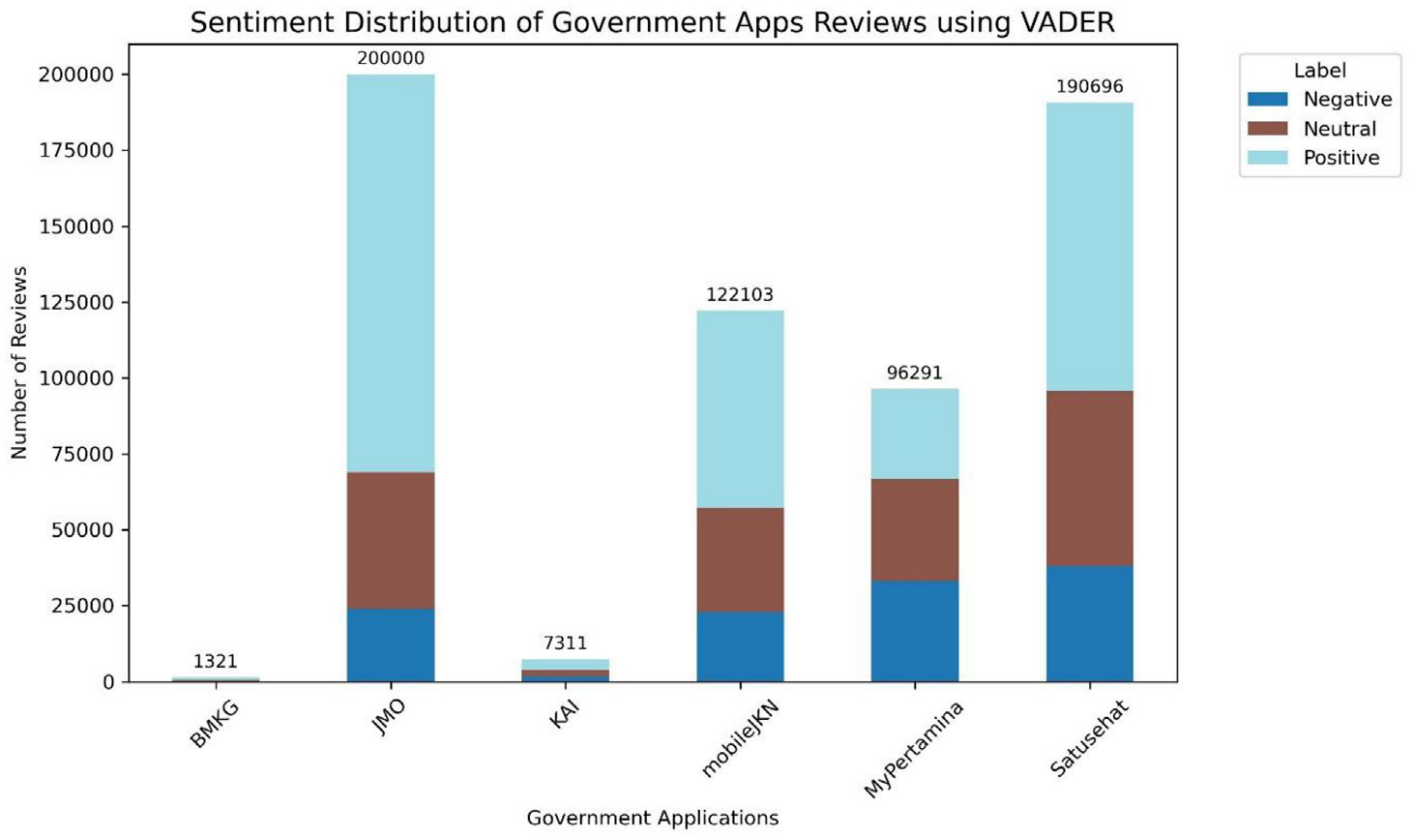
Sentiment distribution using VADER for government-related applications.

**Fig. 7:**
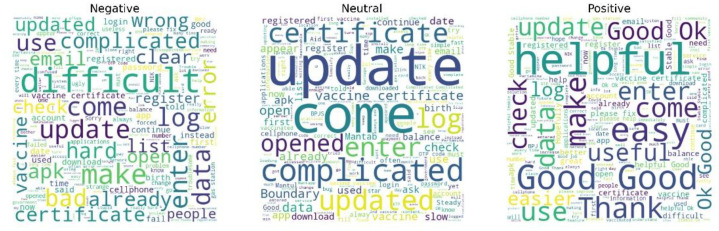
Word Cloud group by label sentiment from all applications in English translation.

Table 8 presents the distribution label generated by VADER. The labeling results show differences in sentiment distribution for each class between VADER-based labeling and ratings in reviews of six government applications. The differences in sentiment labeling results between VADER and ratings are primarily influenced by the fundamental differences in their methodologies. Star ratings reflect users' explicit expressions through numerical choices (e.g., 1–5 stars), which tend to be polarizing: users rate extreme ratings (very satisfied or very dissatisfied) more often than middle ratings, resulting in a distribution dominated by positive and negative categories. In contrast, VADER analyzes review text using a sentiment lexicon, where each word is weighted by positive or negative valence. The total score is then normalized to a compound score (–1 to +1). Fig. 6 visualizes the distribution of six government-related apps based on Table 8. The word cloud of dataset review users in English with VADER Label can be seen in Fig. 7. Although the distribution of records across sentiment categories differs from the rating-based labels, the words that appear largely represent the same themes and issues.

**Table 8:** Distribution of sentiment using VADER for each application.

Government Apps	Label			Total
	Negative	Neutral	Positive	
BMKG	49	523	749	1321
JMO	23986	45021	130993	200000
KAI	1608	2234	3469	7311
MobileJKN	22876	34367	64860	122103
MyPertamina	33201	33549	29541	96291
Satusehat	38013	57635	95048	190696
**Total**	119733	173329	324660	617722

**Fig. 8:**
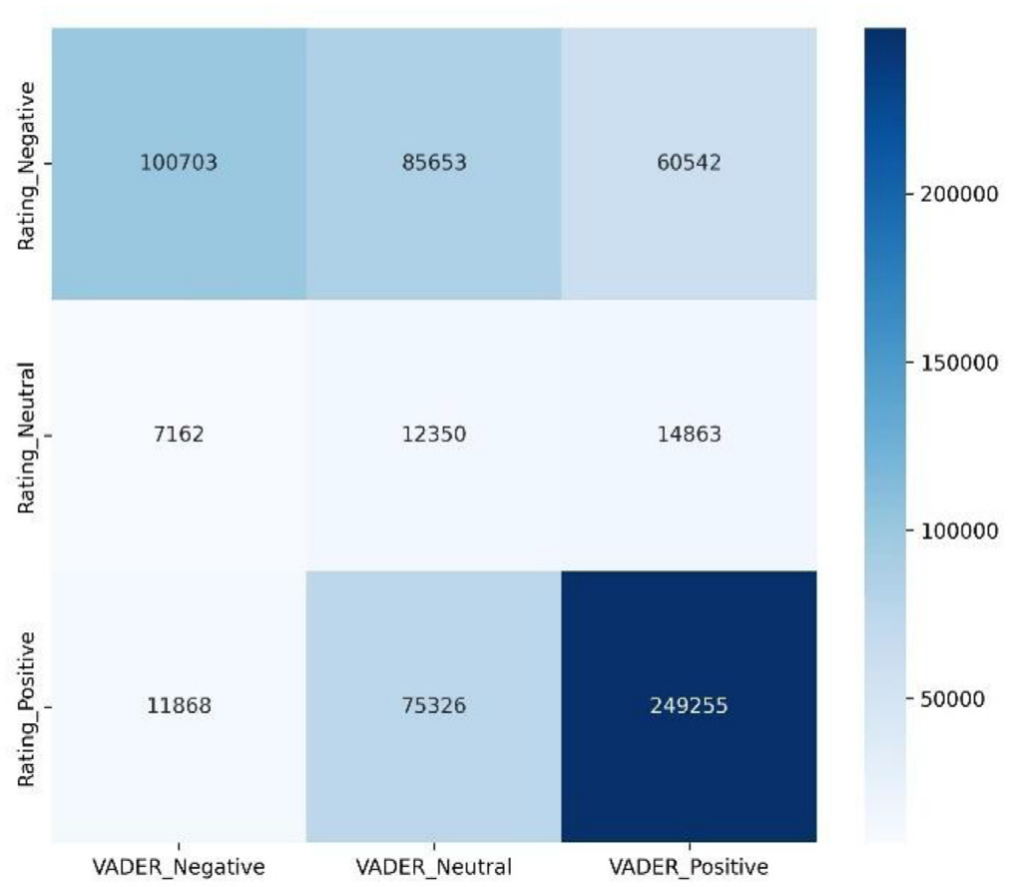
Confusion matrix of agreement labels using Cohen’s kappa.

To further assess the relationship between these two labeling methods, an agreement analysis was conducted to evaluate the consistency between the rating based and VADER based sentiment labels. The result indicated a moderate level of agreement, with Cohen’s kappa score of 0.33 and overall label matching rate of 58.65%. As shown in Fig. 8, most discrepancies occurred in the neutral label, where the VADER model frequently classified rating-labeled negative review as neutral. This pattern suggests that the VADER model adopts a more conservative polarity threshold, especially when dealing with review containing mixed emotional tones. A comparative evaluation of two classical machine learning models, namely Random Forest and LinearSVC, was conducted to establish baseline benchmark results, as shown in Table 9. Both models were trained using TF-IDF features with a maximum of 5000 features. No extensive hyperparameter tuning was performed, and default model configurations were used to provide a fair and reproducible baseline for comparison.

**Table 9:** Performance evalution of machine learning models using IGAR Dataset.

App	Lang	Accuracy		Precision		Recall		F1-Score	
		RF	LinearSVC	RF	LinearSVC	RF	LinearSVC	RF	LinearSVC
Satusehat	ID	0.87	0.87	0.87	0.87	0.87	0.87	0.87	0.87
	EN	0.83	0.83	0.80	0.80	0.83	0.83	0.80	0.81
BMKG	ID	0.87	0.88	0.88	0.88	0.87	0.88	0.86	0.87
	EN	0.85	0.85	0.79	0.80	0.85	0.85	0.81	0.82
KAI	ID	0.82	0.81	0.82	0.81	0.82	0.81	0.82	0.81
	EN	0.78	0.78	0.73	0.75	0.78	0.78	0.74	0.76
MyPertamina	ID	0.87	0.88	0.87	0.88	0.87	0.88	0.87	0.88
	EN	0.85	0.90	0.82	0.87	0.65	0.90	0.82	0.88
MobileJKN	ID	0.88	0.89	0.88	0.89	0.88	0.89	0.88	0.89
	EN	0.85	0.86	0.82	0.82	0.85	0.86	0.82	0.83
JMO	ID	0.92	0.92	0.92	0.92	0.92	0.92	0.92	0.92
	EN	0.89	0.89	0.87	0.87	0.89	0.89	0.88	0.88

## Limitations

The user reviews of government-related applications were limited to Google Play Store for six well-known applications: Mobile JKN, MyPertamina, KAI, JMO, Satusehat, and BMKG. Since reviews from alternative platforms (e.g., iOS App Store or social media) were not considered, the dataset may suffer from limited representativeness and potential platform bias. As a result, it might not provide a complete picture of user sentiment toward government-related applications in Indonesia.

The dataset shows a considerable class imbalance, with positive reviews accounting for about half of the total records. In addition, the distribution of reviews across applications is uneven, further contributing to imbalance issues. To address this challenge, several potential strategies can be considered in future model development. For instance, class weighting can be applied to assign higher importance to minority classes during training, thereby reducing bias toward the dominant class. In addition, resampling techniques such as oversampling the minority class or undersampling the majority class may be employed to achieve a more balanced distribution. Furthermore, the use of robust evaluation metrics, such as macro-averaged F1-score, is recommended to ensure fair performance assessment across all classes.

Even though sentiment labels were assigned using ratings and automatically generated with VADER, the subjective nature of sentiment may lead to misclassification, especially in instances of sarcasm, ambiguity, or mixed opinions. In addition, the sentiment labels were derived directly from user-provided ratings, where each review corresponds to a single user’s evaluation. As a result, no multiple annotators are available for the same instance.

Finally, reviews were primarily collected in Indonesian; however, some records were written in local languages and many also contained slang expressions [20]. This study intentionally preserves the original form of user-generated text without normalization to reflect authentic language usage in real-world digital environments. From an ethical perspective, the use of informal language, regional expressions, and slang may introduce interpretation bias, as sentiment can vary depending on cultural and contextual understanding. Nevertheless, preserving such characteristics is important to avoid over-sanitizing the data and to maintain its ecological validity. Furthermore, since the user reviews were translated automatically using Google translate, some contents were missing in translation due to the limitation of tools.

## Ethics Statement

The authors have read and followed the ethical requirements for publication in Data in Brief and confirm that the current work does not involve human subjects or animal experiments. The data collected was publicly available and completely anonymous, no personal or sensitive information is included, and there are no ethical concerns regarding privacy or data usage associated with this study.

## CRediT authorship contribution statement

**Mahmud Isnan:** Conceptualization, Methodology, Software, Investigation, Writing – original draft. **Bens Pardamean:** Validation, Writing – review & editing, Supervision.

## Data Availability

Mendeley DataIGAR: Indonesian Government App Review Dataset (Original data).

## References

[1] Isnan M., Pardamean B. (2025). IGAR: Indonesian government app review dataset. Mendeley Data.

[2] Tejedo-Romero F., Araujo J.F.F.E., Tejada Á., Ramírez Y. (2022). E-government mechanisms to enhance the participation of citizens and society: exploratory analysis through the dimension of municipalities. Technol. Soc..

[3] Rachmawati R., Sari A.D., Sukawan H.A.R., Widhyastana I.M.A., Ghiffari R.A. (2021). The use of ICT-based applications to support the implementation of smart cities during the COVID-19 pandemic in Indonesia. Infrastruct. (Basel).

[4] Mola S.A.S., Widiastuti T., Roma R.V.K.I.O., Karnyoto A.S., Pardamean B. (2024). Sentiment analysis: indonesia Netflix user’s comment using multiple lexicon-based dictionaries, 2024 International Conference on Intelligent Cybernetics Technology and Applications. ICICyTA.

[5] Yasin A., Fatima R., Ghazi A.N., Wei Z. (2024). Python data odyssey: mining user feedback from google play store. Data Brief..

[6] Hutto C., Gilbert E. (2014). Proceedings of the International AAAI Conference on Web and Social Media.

[7] Abdulrahman A.O., Othman S.I., Yasin G.B., Ali M.S. (2025). A dataset for classifying phrases and sentences into statements, questions, or exclamations based on sound pitch. Data Brief..

[8] Isnan M., Elwirehardja G.N., Pardamean B. (2023). Sentiment analysis for TikTok review using VADER Sentiment and SVM model. Procedia Comput. Sci..

[9] Pham K., Rao Kathala K.C., Palakurthi S. (2025). Reddit sentiment analysis on the impact of AI using VADER, TextBlob, and BERT. Procedia Comput. Sci..

[10] Soni J., Mathur K. (2023). Proceedings of the 3rd International Conference on Innovative Mechanisms for Industry Applications (ICIMIA).

[11] Arunmozhi M., Sunitha R., Rajalakshmi D., Mohan E S. (2025). MADTRAS: dataset for aspect-based sentiment analysis of movie reviews in Tamil. Data Brief..

[12] Sutoyo E., Permana M.C. (2025). Enhancing telemedicine service quality through sentiment analysis of user review dataset in Indonesia. Data Brief..

[13] European Commission (2019). High-Level Expert Group on Artificial Intelligence.

[14] Al-Natour S., Turetken O. (2020). A comparative assessment of sentiment analysis and star ratings for consumer reviews. Int. J. Inf. Manage.

[15] Bigne E., Ruiz C., Perez-Cabañero C., Cuenca A. (2023). Are customer star ratings and sentiments aligned? A deep learning study of the customer service experience in tourism destinations. Serv. Bus..

[16] Alahmary R., Al-Dossari H. (2023). A semiautomatic annotation approach for sentiment analysis. J. Inf. Sci..

[17] Sadiq S., Umer M., Ullah S., Mirjalili S., Rupapara V., Nappi M. (2021). Discrepancy detection between actual user reviews and numeric ratings of Google App store using deep learning. Expert. Syst. Appl..

[18] Goodhew S.C., Edwards M. (2022). Don’t look now! emotion-induced blindness: the interplay between emotion and attention. Atten. Percept. Psychophys..

[19] Islam M.S., Kabir M.N., Ghani N.A., Zamli K.Z., Zulkifli N.S.A., Rahman M.M., Moni M.A. (2024). Challenges and future in deep learning for sentiment analysis: a comprehensive review and a proposed novel hybrid approach. Artif. Intell. Rev..

[20] Rahutomo Anderies R., Pardamean B. (2021). Computer Science and Artificial Intelligence (ICCSAI).

